# Tobacco-Free Schools in Practice: Policy Presence and Enforcement in Baltimore Schools

**DOI:** 10.3390/arm93040028

**Published:** 2025-08-05

**Authors:** Chidubem Egboluche, Rifath Ara Alam Barsha, Shervin Assari, Michelle Mercure, Marc Laveau, Oluwatosin Olateju, Payam Sheikhattari

**Affiliations:** 1 School of Community Health & Policy, Morgan State University, Baltimore, MD 21251, USA; chegb4@morgan.edu (C.E.); payam.sheikhattari@morgan.edu (P.S.); 2 University of Mississippi Medical Center, Jackson, MS 39216, USA; rbarsha@umc.edu; 3 Department of Internal Medicine, Charles R. Drew University of Medicine and Science, Los Angeles, CA 90059, USA; 4 Nationwide Tobacco Programs, American Lung Association, Chicago, IL 60601, USA; michelle.mercure@lung.org; 5 Baltimore City Public Schools, Baltimore, MD 21202, USA; malaveau@bcps.k12.md.us; 6 Helene Fuld School of Nursing, Coppin State University, Baltimore, MD 21216, USA; oolateju@coppin.edu

**Keywords:** school-based tobacco control, youth tobacco use, policy enforcement, urban schools, Baltimore City, tobacco prevention, school policies, policy implementation, smoke-free schools, vape-free schools, health promotion, adolescent health, public health policy, school environment, health disparities

## Abstract

**Highlights:**

**What are the main findings?**

- While many policies existed across the 20 participating schools, their enforcement was widely inconsistent The most present and enforced policies are the ones that require little or no funding/resources.

**What is the implication of the main finding?**

- Greater attention may be needed to support policy implementation and to reduce variability in school-level practices.policies that require minimal funding are more likely to be enforced, indicating that resource availability influences implementation.

**Abstract:**

**Background:** School-based tobacco control policies are critical for preventing youth tobacco use. While many districts adopt formal policies to create smoke- and vape-free environments, the degree to which these policies are enforced at the school level may vary, influencing their effectiveness. Little is known about how consistently such policies are implemented across schools within urban school districts. Objectives: This study aimed to examine the existence and enforcement of school-level tobacco control policies in an urban public school system, using Baltimore City schools as a case example. **Methods:** We conducted a survey of school personnel from 20 high schools in Baltimore City in 2024. The survey instrument assessed the presence and enforcement of policies related to tobacco use prevention, communication, signage, disciplinary actions, and institutional support. Descriptive statistics (frequencies and percentages) were used to summarize responses. Spearman correlations were also used for bivariate correlations. Additional school-level and neighborhood-level contextual data were collected from the internet (neighborhood socioeconomic status and school performance). **Results:** While many policies existed across the 20 participating schools, their enforcement was widely inconsistent. Most schools reported the existence of policies prohibiting tobacco use in school buildings (60%) and vehicles (55%). However, few schools had visible tobacco-free signage (35%) or offered cessation programs (15%). Communication of policies to students (70%) and staff (65%) was the most commonly enforced aspect of tobacco control policies. **Conclusions:** Findings suggest that while tobacco control policies may be adopted across urban school systems, their enforcement at the school level remains uneven. Greater attention may be needed to support policy implementation and to reduce variability in school-level practices. Baltimore City serves as a useful case study to understand these challenges and identify opportunities for strengthening school-based tobacco prevention efforts.

## 1. Introduction

Preventing tobacco use during adolescence remains a cornerstone of public health efforts, as most adult tobacco users initiate the behavior during their youth [1,2,3,4]. Schools are uniquely positioned to contribute to these efforts, not only by educating students about the harms of tobacco use but also by enforcing policies that create smoke- and vape-free environments [5,6,7,8]. Effective school-based tobacco prevention strategies often involve clear signage, comprehensive education, student screening, enforcement mechanisms, and communication of policies to students, staff, and families [5].

A growing body of research shows that tobacco control policies can reduce youth smoking initiation, decrease exposure to secondhand smoke, and promote cessation among those who already use tobacco [9,10,11,12]. Policies that create tobacco-free school environments such as banning smoking on school property, integrating tobacco education into curricula, and offering cessation support have been associated with more favorable student health behaviors and outcomes such as lower tobacco use [9,13,14]. These interventions not only support individual behavior change but also shape broader norms around tobacco use. The mere presence of a policy may be insufficient to achieve meaningful outcomes, as studies suggest that its effectiveness largely depends on the degree of enforcement, frequency of communication, and consistent monitoring and implementation [15,16,17,18].

Inconsistent enforcement may lead to confusion, normalization of rule-breaking, and reduced trust in institutional authority [15]. Conversely, strong and consistent enforcement may signal that tobacco use is taken seriously, thereby reinforcing prevention messages [15,18,19,20]. Many states allocate funding for tobacco control that falls short of recommended levels, particularly for school-based prevention initiatives [21]. This inadequate investment can impede the implementation and enforcement of tobacco-free policies, limit student education on tobacco-related risks, and restrict access to cessation support services. Understanding this gap between policy existence and policy enforcement is especially important in school settings, where policies may be unevenly applied due to differences in resources, leadership priorities, or staff capacity. While district-level mandates set the framework, their success often depends on how individual schools translate and implement them on the ground. This gap represents a potential leverage point for strengthening tobacco prevention efforts within educational systems.

To illustrate this issue, Baltimore City schools serve as a case study, having adopted a range of smoke- and vape-free school environment policies [22], offering an opportunity to explore their implementation at the school level. Over the past few decades, tobacco use in the general US population has been on a downward trend from 42.4% in 1965 to 12.5% in 2020 [23]. Although this decline is one of the greatest successes of public health, demographic disparities in smoking persist [23,24]. Baltimore City has exhibited higher-than-average rates of youth tobacco use, including both combustible cigarettes, cigars, and smokeless tobacco compared to state benchmarks [25]. These elevated rates are compounded by structural inequalities, including high poverty rates, concentrated disadvantages, and limited access to youth-centered preventive services [26]. At the same time, Baltimore City has been a site of policy innovation, implementing local ordinances that restrict tobacco advertising near schools, and raising the minimum sales age. However, enforcement of these policies remains inconsistent, often challenged by limited municipal resources [27], industry pushbacks [28,29], and uneven retailer compliance [29]. Studying tobacco control efforts in Baltimore City can therefore yield important insights into the barriers and opportunities for reducing youth tobacco initiation in urban settings shaped by inequality and resilience.

### Aims

This study aims to assess the extent to which tobacco control policies are present and enforced in Baltimore City public schools (BCPS), with attention to different domains such as policy communication, disciplinary action, education, signage, and institutional support.

## 2. Methods

### 2.1. Design and Setting

This is a cross-sectional survey of schools at Baltimore City public schools (BCPS), USA. BCPS is a large urban school district in Maryland serving over 77,000 students [30]. The district is predominantly African American (69.6%) and economically disadvantaged, with most students qualifying for free or reduced-price meals [30]. BCPS has formally adopted smoke- and vape-free environment policies; however, school-level enforcement may vary.

### 2.2. Ethics

This study was approved by the Office of Achievement and Accountability (OAA) Institutional Review Board (IRB00008794). This descriptive cross-sectional study was conducted in Baltimore City public schools at the end of 2024.

### 2.3. Participants

We invited staff members from the 34 high schools in BCPS through email, and 20 schools indicated interest to participate in the survey. The participants included 20 Community School Coordinators (CSCs), selected based on their familiarity with school policies and operations. Each participant provided information on their respective school’s tobacco-related policies and their enforcement. One respondent per school completed the survey through their respective emails.

### 2.4. Survey Instrument

We developed a structured survey tool with Kobo Toolbox, which included items covering a broad set of tobacco control policy domains relevant to schools. The survey instrument was developed by extracting policy items from formally adopted smoke- and vape-free environment policies published on the BCPS Board website. These items were then thematically organized into four policy domains. The instrument was designed to assess both the existence and enforcement of each policy at the school level. As shown in Table 1, items were grouped into thematic areas: (1) communication of policies (e.g., whether smoke- and vape-free policies are shared with students, staff, parents, or during enrollment), (2) prohibition policies (e.g., banning tobacco use on school grounds, in school vehicles, or near entrances/playgrounds), (3) disciplinary enforcement (e.g., student code of conduct, actions for staff or visitor violations), and (4) school-level support (e.g., signage, tobacco education programs, cessation initiatives, screening for use, and resource allocation).

Each item asked whether a given policy existed at the school and whether it was being actively enforced. The format of most questions was yes/no. The survey also included brief questions about the respondent’s role and the school type (e.g., public, charter, grade levels served).

### 2.5. Additional Data Source

Additional contextual data about schools such as median income of school zip code, education level of zip code, and school-level characteristics such as overall performance, math proficiency, poverty rate, and graduation rate were supplemented through the Baltimore City public schools website [31] and United States Census Bureau [32]. Variables such as race, overall school performance score, 5-star school ranking, graduation rate, and poverty rate were included to capture institutional factors that may influence policy adoption and implementation. Neighborhood-level characteristics, including median household income and educational attainment within the school zip code, were incorporated to reflect the broader socioeconomic context in which schools operate. These variables were chosen to examine whether disparities in policy presence and enforcement correspond to variations in school performance, demographics, and surrounding community conditions.

### 2.6. Statistical Approach

The Stata SE 16 software was used for statistical analysis. Descriptive statistics, including medians, frequencies, and percentages, were used to summarize school-level responses. For each policy item, we calculated the number and percentage of schools (out of 20) that reported having and enforcing the policy. Additionally, Spearman’s rank-order correlation (number of correlations = 24) was used to examine relationships between select school characteristics and policy enforcement variables. Given the exploratory nature of this study and the small sample size, a stricter significance threshold (*p* < 0.01) was applied to reduce the risk of false positives. Formal correction methods (e.g., Bonferroni’s) were not used, as they may be overly conservative in exploratory analyses and increase the risk of overlooking potentially meaningful associations. No multivariate modeling was conducted, as the goal was to explore patterns of policy implementation rather than to test causal hypotheses.

## 3. Results

### 3.1. School Characteristics

As Table 2 depicts, the 20 participating Baltimore City public high schools varied in academic performance, socioeconomic context, and demographics. Each year, the Maryland Report Card includes a star rating for each school in the state. Schools earn from one to five stars yearly based on critical academic, college readiness, and school quality and student success measures [31]. Most BCPS had 2–3-star ratings under the district’s Five-Star Ranking System, with overall performance scores ranging from 21.6% to 55.6%. Graduation rates ranged from 10% to ≥95%. All were Title-1-eligible, with poverty rates between 42% and 80%. Title-1-eligible schools refer to schools that qualify for federal funding due to having a high percentage (at least 40%) of students from low-income families. Academic outcomes were generally low, with most schools reporting ≤5% math proficiency. The racial composition was predominantly African American, with most schools reporting African American student populations above 85%. Median household incomes in school ZIP codes ranged from USD 37,207 to USD 87,669, and the percentage of adults with a bachelor’s degree or higher ranged from 12.40% to 60.4%.

### 3.2. Policy Enforcement

As Table 3 shows, the survey findings revealed varying levels of enforcement across tobacco policy domains in the 20 participating Baltimore City schools. Overall, policies related to communication were the most consistently reported, while education, screening, and resource allocation were among the least implemented.

Communication of tobacco policies appeared to be a relatively strong area of enforcement. Most schools reported informing students (70%) and staff (65%) about the smoke- and vape-free policy, and about half (50%) extended this communication to parents or guardians and included the information at the beginning of the school year. Fewer schools (30%) consistently communicated the policy at enrollment or every semester.

In contrast, policy enforcement related to educational programming and institutional support was sparse. Very few schools had tobacco education embedded in the curriculum (10%), cessation support programs available (15%), or budgetary allocations for prevention (10%). Similarly, only a small number screened students for tobacco use (20%) or reported having a dedicated interdepartmental team for tobacco prevention efforts (15%).

Schools were more likely to report enforcing prohibition policies, especially those concerning tobacco use in school-owned buildings and vehicles (60% and 55%, respectively). Prohibitions near entrances, playgrounds, and other school-adjacent areas were less commonly (40%) enforced. Similarly, disciplinary responses to violations, such consequences for staff and visitors, and actions outlined in student codes of conduct were reported by just over half of the schools (55%, 55%, and 60%, respectively).

Fewer schools (35%) reported visible no-smoking or no-vaping signage, and only half (50%) noted that a district-level policy had been formally adopted. These findings suggest that while the policy framework may be present, its visibility and consistency in enforcement remain uneven across school settings.

The correlation analyses across all the school demographic indicators and policy enforcement domains identified just one significant association. Specifically, median income of neighborhood is inversely correlated with communication of the smoke- and vape-free policy at student enrollment (ρ = –0.57, *p* = 0.008). The result also showed correlation within domains. Among academic performance indicators, the strongest positive correlations were observed between the district’s 5-star school rating and overall performance score (ρ = 0.92, *p* < 0.001), between the 5-star rating and graduation rate (ρ = 0.90, *p* < 0.001), and between overall performance score and graduation rate (ρ = 0.89, *p* < 0.001).

Tobacco policy communication variables exhibited very strong inter-correlations: for example, communicating the smoke- and vape-free policy to students was positively associated with communicating to staff (ρ = 0.75, *p* < 0.001), to parents (ρ = 0.75, *p* < 0.001), at enrollment (ρ = 0.65, *p* = 0.002), every semester (ρ = 0.60, *p* = 0.005), and at the beginning of the school year (ρ = 0.74, *p* < 0.001). Communication to staff correlated with communication to parents (ρ = 0.85, *p* < 0.001), at enrollment (ρ = 0.57, *p* = 0.008), every semester (ρ = 0.73, *p* < 0.001), and at the beginning of the school year (ρ = 0.86, *p* < 0.001). Communication to parents was correlated with communication at enrollment (ρ = 0.71, *p* = 0.001), every semester (ρ = 0.83, *p* < 0.001), and at the beginning of the school year (ρ = 0.89, *p* < 0.001).

Enforcement infrastructure measures also clustered: allocation of funds for prevention programs correlated with interdepartmental team support (ρ = 0.80, *p* < 0.001). Enforcement of vehicle prohibitions correlated strongly with perimeter prohibitions (ρ = 0.79, *p* < 0.001) and visitor warnings (ρ = 0.54, *p* = 0.013). Among the disciplinary measures themselves, student code sanctions correlated with both staff sanctions (ρ = 0.73, *p* < 0.001) and visitor warnings (ρ = 0.82, *p* < 0.001), and staff sanctions correlated with visitor warnings (ρ = 0.76, *p* < 0.001).

## 4. Discussion

This study aimed to examine the presence and enforcement of tobacco control policies across 20 schools in Baltimore City. While many school districts adopt formal policies to create smoke- and vape-free environments, we sought to understand the degree to which these policies are actively implemented on the ground.

Our findings suggest variability in enforcement across clusters of tobacco-related policies. Communication of smoke- and vape-free policies emerged as the most consistently enforced domain. Most schools reported informing students (70%) and staff (65%) about these policies, and roughly half communicated them to parents and guardians during enrollment. These patterns may reflect a general prioritization of policy awareness, even if other supports are not in place [6,33,34], as communication typically requires minimal resources, an approach likely influenced by the resource constraints faced by schools in underserved communities. A study by Barbero et al. (2013) conducted in St. Louis, Missouri, found that policy awareness scored highest on the tobacco-free environment domain [33].

In contrast, screening, education, and prevention programming showed the lowest levels of enforcement. Only a small number of schools reported having tobacco education integrated into the curriculum (10%), cessation programs available (15%), or student screening practices (20%). Very few schools reported allocating any funds for tobacco prevention. These gaps suggest that while schools may be aware of policies, they may lack the resources, personnel, or support to provide comprehensive prevention services [27,35,36]. Policies related to prohibition and disciplinary enforcement were moderately enforced. For instance, 60% of schools reported prohibiting tobacco use in school buildings and grounds, while about half enforced policies for tobacco use in school vehicles or near school entrances. Around 55–60% of schools used student codes of conduct, staff reprimands, or visitor warnings to enforce violations. These levels suggest that policies tied to school safety or discipline frameworks may be more likely to be enforced than those requiring health education or resource allocation as these schools face unique challenges, which include staff shortage, burnout, and limited access to funding [6,14,27,37].

Only 10% of schools had any tobacco education in the curriculum or funds allocated for prevention. This pattern of selective enforcement underscores a broader challenge identified in the literature. As documented in prior studies, limited staffing, lack of professional development, and competing academic priorities often hinder the implementation of holistic tobacco prevention strategies in under-resourced schools [38,39,40].

The inverse associations between socioeconomic indicators (median income of neighborhood) and communication of smoke- and vape-free policies may reflect a paradox in how public health messaging is prioritized across educational contexts [41]. Schools in higher-income neighborhoods may not prioritize proactive school tobacco policy communication to parents, possibly due to assumptions of existing awareness or lower perceived risk among their populations. This phenomenon aligns with findings of studies showing that higher-socioeconomic-status groups often have greater baseline health knowledge, leading institutions to under-communicate policies under the assumption that these populations are already informed [42]. Supportive informal norms and social capital may be more prevalent and influential in high-socioeconomic-status environments regarding healthy behaviors [43]. These disparities suggest that policy communication may not be uniformly valued across contexts but may instead be strategically or unconsciously modulated by school perceptions of community health literacy and behavioral risk.

### 4.1. Limitations

This study has several limitations. First, the small sample size (n = 20 schools) limits generalizability within Baltimore City or similar urban districts. Second, responses were self-reported and may be subject to recall or social desirability bias. The survey relied on single informants per school, which may not fully capture the variation in enforcement practices. Additionally, there is a potential for systematic bias in who chose to participate. Schools with more robust policies or administrative engagement may have been more likely to participate. Another potential bias would be the sampling bias. There would be a limited generalizability of the findings due to the study’s setting in a single, predominantly African American and economically disadvantaged urban school district. The findings may not be representative of other populations or school districts with different demographic or socioeconomic profiles. Reporting bias is a concern, particularly because key variables, including childhood abuse, depressive symptoms, and education, were based on self-reporting. Respondents may underreport traumatic experiences or mental health symptoms due to stigma, memory limitations, or social desirability, which could lead to measurement error or attenuation of observed associations. While the analyses were exploratory in nature, the number of comparisons increases the risk of type I error. The study did not measure any relevant endpoints, such as tobacco use prevalence among students, as the absence of such data makes it impossible to evaluate the real-world impact of the policies being studied. Despite these limitations, the large and diverse international sample strengthens the robustness of the observed patterns and offers valuable insights into resilience mechanisms across contexts.

### 4.2. Implications

These findings raise important considerations for both policy and practice. While district-level tobacco control policies may be adopted with good intent, their uneven enforcement may weaken their overall impact. Gaps in communication, education, and enforcement may also send mixed messages to students, undermining the creation of a truly tobacco-free environment. These findings underscore the impact of limited resources and socioeconomic inequalities on policy enforcement at schools in underserved neighborhoods. Strengthening implementation, particularly in areas such as prevention programming and resource allocation, may help reinforce policy goals.

### 4.3. Future Research

Future studies should explore the reasons behind enforcement variability, including administrative capacity, resource constraints, staff training, and perceptions of policy relevance. It would also be valuable to include student perspectives, direct observations, and longitudinal data to assess how enforcement patterns relate to youth tobacco use over time. Expanding this work to include multiple cities or rural districts could also offer insight into place-based implementation challenges.

### 4.4. Future Interventions for Schools

To support more comprehensive implementation, schools may benefit from district-level support that goes beyond policy mandates. This could include dedicated funding for tobacco education and cessation programs, training for school staff on enforcement protocols, and standardized procedures for policy communication. Additionally, interdisciplinary teams within schools may enhance coordination and accountability across departments such as health, administration, and student services.

## 5. Conclusions

In summary, this study highlights significant variation in how school tobacco control policies are enforced within an urban school district. Communication policies were more consistently reported, while education, prevention, and institutional support were limited. These patterns suggest that implementation, not just adoption, deserves greater attention if school-based tobacco control efforts are to achieve their full potential. While Baltimore City serves as a useful case study, the implications likely extend to many other districts working to protect youth from tobacco-related harm.

## Figures and Tables

**Table 1:** Questions used in the survey.

Prohibition policies
Sale or use of tobacco in any form is prohibited in any vehicle owned, leased, or contracted by the Board, including school buses.
Sale or use of tobacco in any form including electronic cigarettes or vaping products in all Board-owned or -leased buildings and on all school grounds is prohibited at all times.
Smoking is prohibited in or within 50 feet of any City Schools playground, school entrance, window, or air intake vent regardless of whether that is on school, public, or other property, schoolyard, or athletic facility.
Communication of policies
The smoke- and vape-free school environment policy is communicated to students.
The smoke- and vape-free school environment policy is communicated to teachers/staff.
The smoke- and vape-free school environment policy is communicated to parents/guardian.
The smoke- and vape-free school environment policy is communicated at enrollment.
The smoke- and vape-free school environment policy is communicated every semester.
The smoke- and vape-free school environment policy is communicated at the beginning of a school year.
School-level support
The school screens students for tobacco use at any time.
The school allocates some funds to tobacco use prevention programs.
An interdepartmental team supports tobacco use prevention initiatives and policies in the school.
There are “No-Smoking/Vaping” signs around the school environment/building.
There is a tobacco education program in the school/school curriculum.
There is a tobacco cessation program/initiative in the school.
Baltimore City School board has a smoke- and vape-free school environment policy.
Disciplinary enforcement
The student code of conduct disciplinary action is used for students violating the smoke- and vape-free school environment policy.
The school reprimands first offenders and suspends/dismisses repeat offenders for teacher/staff violation of the smoke- and vape-free school environment policy.
The school warns, and as appropriate, requests to leave the school building/school grounds for visitor violation of the smoke- and vape-free school environment policy.

**Table 2:** School characteristics.

Demographics (n = 20)	Median	Range	
		Minimum	Maximum
Race (%)			
African American	85.0	30.0	95.0
Hispanic	8.0	5.0	59.0
White	5.0	5.0	17.0
Asian	5.0	5.0	5.0
American Indian	5.0	5.0	5.0
Pacific Islander	5.0	5.0	5.0
Multiracial	5.0	5.0	5.0
5-Star School Ranking	2.0	1.0	4.0
Overall Performance Score (%)	43.5	21.6	68.9
Graduation Rate (%)	77.5	10.0	95.0
% Poverty rate (Title 1 eligibility)	71.0	42.0	82.0
School Neighborhood Data			
Income of School Zip code (Median Household)	USD 48,837.5	USD 37.207.0	USD 87,669.0
% Education level of School Zip code (Bachelors’ Degree or Higher)	23.7	12.4	60.8

**Table 3:** Policy existence and enforcement in Baltimore schools.

Policy Domain and Item	n/20	%
**Policy Existence**		
Prohibition Policies		
Sale/use prohibited in Board buildings and grounds	12	60
Prohibited in vehicles (including buses)	11	55
Smoking prohibited within 50 feet of school/playgrounds	8	40
Existence of citywide school board smoke-/vape-free policy	10	50
Physical Signs		
No-Smoking/Vaping signs posted	7	35
Screening, Education, and Prevention Support		
School screens students for tobacco use	4	20
Tobacco cessation program available	3	15
Interdepartmental team supports prevention	3	15
Tobacco education in curriculum	2	10
Funds allocated for tobacco prevention	2	10
**Policy Enforcement**		
Communication of Smoke-Free Policy		
Smoke-free policies communicated to students	14	70
Smoke-free policies communicated to teachers/staff	13	65
Smoke-free policies communicated to parents/guardians	10	50
Smoke-free policies communicated at beginning of school year	10	50
Smoke-free policies communicated at enrollment	6	30
Smoke-free policies communicated every semester	6	30
Enforcement and Disciplinary Action		
Student code of conduct used for violations	12	60
Staff reprimanded/suspended for violations	11	55
Visitors warned or removed for violations	11	55

## Data Availability

Data is available upon request to the authors.
